# Deep learning image reconstruction optimizes coronary artery calcium quantification

**DOI:** 10.3389/fcvm.2026.1639920

**Published:** 2026-03-31

**Authors:** Tao Zhou, Ming Liu, Ting Wu, Min Zhang, Jianjun Dong, Zhuangfei Ma, Ying Li, Xinru Ba

**Affiliations:** 1 Department of Radiology, People’s Hospital Affiliated to Shandong First Medical University (Jinan City People’s Hospital), Jinan, Shandong Province, China; 2 Department of Radiology, Yantai Yuhuangding Hospital, Yantai, China; 3 Canon Medical System (China), Beijing, China; 4 Department of Radiology, Yantaishan Hospital, Yantai, China

**Keywords:** coronary artery calcium, coronary artery disease, deep learning, filtered back projection, hybrid iterative reconstruction

## Abstract

**Objective:**

To investigate the effects of deep learning reconstruction (DLR) on the image quality and quantification of coronary artery calcium (CAC).

**Materials and methods:**

Patients who underwent calcium scoring and coronary CT angiography examinations were retrospectively collected. The images of calcium scoring were reconstructed using filtered back projection (FBP), hybrid iterative reconstruction (HIR) and DLR algorithms. The CT value, image noise, signal-to-noise ratio (SNR) and contrast-to-noise ratio (CNR) of aortic root and left ventricle were compared in three algorithms. Two radiologists scored the subjective image quality using a four-point scale. The quantification of CAC (Agatson score, calcium volume and mass) in FBP, HIR and DLR were calculated by automatic software. The risk classification of CAC were evaluated according to the Agatston score.

**Results:**

In objective image quality, compared with FBP and HIR, DLR significantly reduced image noise and improved SNR (all *p* < 0.05) without changing the CT value of aortic root and left ventricle (all *p* > 0.05). DLR received significantly higher subjective scores (3.80 ± 0.40) than HIR (3.48 ± 0.50) and FBP (2.36 ± 0.48) (both *p* < 0.001). In calcium quantification, the Agatston score, calcium volume and mass were no significant difference among the three algorithms (all *p* > 0.05). In risk classification analysis, DLR reduced the number of reclassification compared with HIR.

**Conclusion:**

DLR enhances the image quality and consistency of CAC quantification compared with FBP and HIR. Besides, DLR reduced risk reclassification relative to HIR.

## Introduction

Coronary artery disease (CAD), as a cause of cardiovascular mortality, requires early risk stratification and precise diagnosis to guide clinical management (1). The coronary artery calcium (CAC) which obtained by non-contrast CT scans could detect atherosclerosis caused by calcification and quantify coronary calcification. This approach addresses limitations of traditional risk assessment while demonstrating cost-effectiveness (2). Filtered back-projection (FBP) algorithm is a conventional reconstruction method used in CT images, but the image noise of FBP images remarkable increases with low radiation dose (3–5). Although hybrid iterative reconstruction (HIR) algorithm has significantly improved image quality in CAC images, challenges remain in accurately identifying microcalcifications (4, 6).

Recently, deep learning reconstruction (DLR) algorithm has been developed for improvement of image quality and diagnostic performance in CT images (7, 8). As a CT image post-processing technique, DLR achieves noise reduction and artifact suppression in the image space by mapping low-dose hybrid IR images to high-dose model-based iterative reconstruction (MBIR) images. Its training adopts a fully supervised approach, using high-dose MBIR images as the target and low-dose hybrid IR images simulated at various dose levels and reconstruction fields of view as the input. The training optimizes a ten-layer deep convolutional residual network by minimizing mean squared error, iteratively converging the loss using the Adaptive Moment Estimation (ADAM) algorithm (9). Therefore, DLR algorithm could enhance the image quality by suppressing artifacts, reducing noise, and improving spatial resolution (10, 11). Many studies have reported that DLR could improve the image quality in coronary CT angiography (12, 13). However, the effects of DLR in CAC remains unknown.

Therefore, this study aims to evaluate the effects of DLR on image quality of CAC and quantification of CAC and validate clinical utility of DLR for CAD risk classification.

## Materials and methods

### Study population

Continuous patients who underwent calcium scoring and coronary CT angiography examinations at our hospital (People's Hospital Affiliated to Shandong First Medical University) for suspected of CAD between September 2024 and March 2025 were retrospectively collected. Inclusion criteria: (1) patients with calcium plaques. (2) over 18 years old. Exclusion criteria: motion artifacts or stent implantation. The study protocol was approved by the institutional ethics committee and written informed consent was taken from all the patients. Sample size estimation of this study was calculated by G*Power software (14).

### CT acquisition

All patients were scanned 320-row CT (Aquilion One Genesis Edition, Canon Medical Systems, Japan). Patients with heart rate > 70 beats/min were given oral beta-blockers prior to the scan. Calcium scoring was scanned with prospective ECG triggering at a 75% R-R interval. Scan parameters were following: 120 kV tube voltage, automatic tube current modulation (standard deviation 40 HU), detector collimation 320 × 0.5 mm, rotation time 0.275 s/circle, field of view 320 mm, matrix size 512 × 512. The scanning range was from the carina to the level of the diaphragm, including the entire heart. Radiation dose including the CT dose index (CTDIvol) and dose length product (DLP) of each patient were recorded. The effective dose (ED) was calculated as ED = DLP×0.014 mSv/(mGy·cm).

### Image reconstruction and analysis

Calcium scoring data were reconstructed using FBP (filtered back projection, FC12), HIR [Adaptive Iterative Dose Reduction (AIDR) 3D, FC12] and DLR [Advanced Intelligent Clear-IQ Engine (AiCE), Cardiac kernel], respectively. All images were reconstructed at a slice thickness/interval of 3.0 mm. The images were transferred to post-processing software (CCSpro, Neusoft Medical) which was extensively trained on FBP and HIR reconstructed images for calcium quantification analysis. The technology first identified and extracted calcified plaques through threshold segmentation and connected component analysis, then it calculated the calcification score accordingly. Simultaneously, it segmented the cardiac tissue and constructed the vascular tree structure based on the segmentation results, which helped delineate vascular pathways. Finally, it automatically completed calcification localization and total calcification measurement by integrating the plaque extraction, cardiac tissue segmentation, and vascular tree construction results. All images were transferred to post-processing workstation (Vitrea Workstation) for image quality evaluation.

### Objective image quality

A radiologist with 7 years work experience in diagnosis of cardiac imaging drew the circle region of interest (ROI) on the aortic root, left ventricle and erector spinae muscle. The area of circle ROI was 100 mm^2^. The mean CT value and standard deviation (SD) of ROI were recorded, signal-to-noise ratio (SNR) and contrast-to-noise ratio (CNR) was calculated by the following formula: $SNR_{aortic\;root}=\frac{CT_{aortic\;root}}{SD_{aortic\;root}},SNR_{left\;ventricle}=\frac{CT_{left\;ventricle}}{SD_{left\;ventricle}},CNR_{aortic\;root}=$ $S\frac{CT_{aortic\;root}-CT_{erector\;spinae\;muscle}}{SD_{erector\;spinae\;muscle}},CNR_{left\;ventricle}=\frac{CT_{left\;ventricle}-CT_{erector\;spinae\;muscle}}{SD_{erector\;spinae\;muscle}}$

### Subjective image quality

Two radiologists (with 10 years work experience in diagnosis of cardiac imaging) blindly evaluated subjective image quality using a 4-point scale: 1 (Poor): Non-diagnostic image quality with indistinct calcium delineation; 2 (Fair): Significant quality degradation but sufficient for diagnostic interpretation; 3 (Good): Mild quality reduction with adequate calcium visualization; 4 (Excellent): Optimal image quality with sharp calcium definition (15, 16).

### Calcium quantification

Two radiologists with 3 and 8 years work experience in diagnosis of cardiac imaging performed two calculations (the minimum one week interval between two calculations) of the Agatston score, calcium volume and mass using a commercial automated calcium analysis software. The software automatically identified coronary arteries by convolutional neural network and calcified lesions by segmentation of pixels with density larger than 130 HU and area larger than 1 mm^2^. The radiologist manually removed the calcified lesions which are not belong to the coronary arteries and recorded the results of Agatston score, calcium volume and mass. All results underwent re-review by a senior radiologist with 10 years work experience in diagnosis of cardiac imaging. Patients were classified into five risk categories: 0 (very low), 1–10 (low), 11–100 (intermediate), 101–400 (high), and >400 (very high) (17, 18).

### Statistical analysis

Statistical analyses were performed with SPSS 21.0. Categorical data were reported as frequency and percentage. Continuous variables were initially analyzed by the Shapiro–Wilk test to verify the data distribution. The normal distribution data were expressed mean ± SD and one-way ANOVA test with Bonferroni correction was performed, while non-normal distribution data were reported as median (Q_1_, Q_3_) and Kruskal–Wallis test were performed. The consistency of the inter-observer image quality score was assessed by Cohen's Kappa test. The Agatston-based risk reclassification is evaluated by weigh Kappa. The degree of agreement was as follows: 0.00–0.20, 0.21–0.40, 0.41–0.60, 0.61–0.80, or 0.81–1.00, indicating slight, fair, moderate, substantial, or almost perfect agreement, respectively. The intra- and inter-observer reproducibility of Agatston score was assessed by intraclass correlation efficient (ICC). *P* < 0.05 was considered statistically significant.

## Results

### Study population

**Figure 1:**
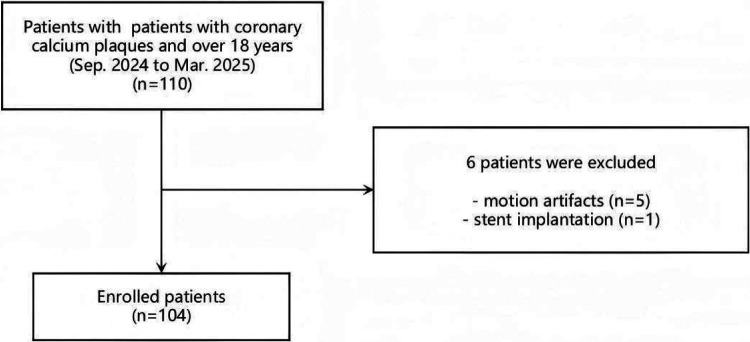
The flowchart of this study.

The sample size estimation of this study was 105. 110 patients were initially collected according to inclusion criteria. Five patients with motion artifacts and one patient with stent implantation were excluded (Figure 1). Finally, 104 patients (63 male, 41 female; mean age 63.32 ± 7.05 years) (Table 1) were included for analysis.

**Table 1:** Characteristics of patients.

Characteristics	Value
Number	104
Age (years)	63.32 ± 7.05
Gender (male/female)	67/43
BMI (kg/m^2^)	23.4 ± 4.2
Heart rate	62.3 ± 5.7
Chest pain	25 (23.6%)
Hypertension	40 (37.7%)
Diabetes	18 (17.0%)
Smoking	23 (21.7%)
CTDIvol (mGy)	10.5 ± 2.8
DLP (mGy.cm)	174.3 ± 45.6
ED (mSv)	2.4 ± 0.6

BMI, body mass index; CTDIvol, the volume CT dose index; DLP, dose length product; ED, effective dose.

### Objective image quality

DLR demonstrated significantly lower image noise and higher SNR and CNR compared to HIR and FBP (all *p* < 0.05, Table 2). HIR showed significantly lower CT value of aortic root and left ventricle (*p* < 0.05, Table 2), however CT value of aortic root and left ventricle of DLR were comparable with those of FBP and HIR (all *p* > 0.05, Table 2).

**Table 2:** Objective image quality of aortic root and left ventricle in FBP, HIR and DLR images.

Location	Measurement parameter	FBP	HIR	DLR	p	p1	p2	p3
Aortic root	CT value	48.90 ± 8.77	46.46 ± 6.97	47.89 ± 5.84	*P* < 0.001	0.017	0.188	0.092
	SD	92.59 ± 19.89	23.88 ± 3.91	18.98 ± 2.68	*p* < 0.001	*p* < 0.001	*p* < 0.001	0.017
	SNR	0.56 ± 0.17	2.01 ± 0.52	2.37 ± 0.50	*p* < 0.001	*p* < 0.001	*p* < 0.001	*p* < 0.001
	CNR	0.08 ± 0.07	0.13 ± 0.03	0.21 ± 0.03	*p* < 0.001	*p* < 0.001	*p* < 0.001	*p* < 0.001
Left ventricle	CT value	51.47 ± 11.75	46.35 ± 10.22	48.73 ± 8.83	0.005	0.003	0.225	0.377
	SD	112.66 ± 35.29	25.36 ± 3.50	17.90 ± 3.51	*p* < 0.001	0.045	*p* < 0.001	0.047
	SNR	0.51 ± 0.20	1.87 ± 0.54	2.88 ± 1.10	*p* < 0.001	*p* < 0.001	*p* < 0.001	*p* < 0.001
	CNR	0.08 ± 0.06	0.13 ± 0.02	0.14 ± 0.07	*p* < 0.001	*p* < 0.001	*p* < 0.001	*p* < 0.001

p1: FBP vs. HIR; p2: FBP vs. DLR; p3: HIR vs. DLR. Data are expressed as mean ± standard deviation.

### Subjective image quality

Two radiologists showed excellent interobserver agreement (kappa = 0.94) in assessment of subjective image quality. DLR received significantly higher subjective scores (3.80 ± 0.40) than HIR (3.48 ± 0.50) and FBP (2.36 ± 0.48) (both *p* < 0.001).

### Calcium quantification

**Figure 2:**
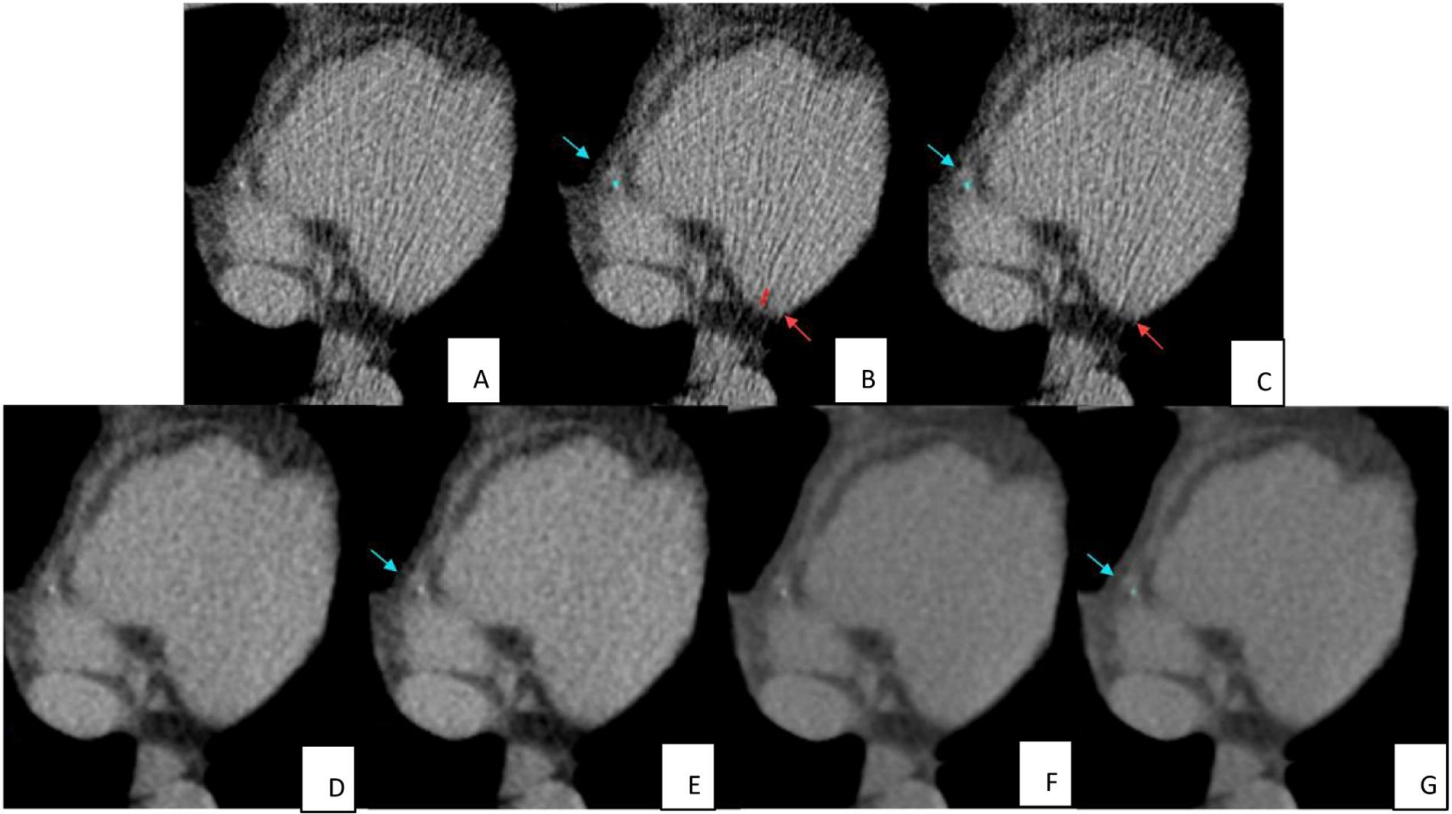
Comparison of CAC images using FBP, HIR, and DLR algorithms. This set of CAC score images demonstrates coronary artery risk stratification as high-risk (FBP, Agatston score 129.47), intermediate-risk (HIR, Agatston score 90.63), and high-risk (DLR, Agatston score 112.04). The pre-processing images [**(A)** for FBP, **(D)** for HIR, **(F)** for DLR] and post-processing results [**(B)** and **(C)** for FBP, **(E)** for HIR, **(G)** for DLR] are displayed for each algorithm, respectively. A small calcification (highlighted by blue arrows) was not detected in the HIR image **(E)** but was identified by both FBP **(B,C)** and DLR images **(G)** Calcification outside coronary artery was detected in the FBP image [red arrow, **(B)**], which was subsequently manual removed **(C)** CAC, coronary artery calcium; FBP, filtered back-projection; HIR, hybrid-iterative reconstruction; DLR, deep learning reconstruction.

The calcium quantification results were presented in Table 3. The Agatston score, calcium volume and mass showed no significant difference among the three reconstruction algorithms (*p* = 0.155, *p* = 0.446 and *p* = 0.069, respectively). The intra- and inter-observer reproducibility of Agatston score were good in three reconstruction algorithms (ICC of FBP > 0.85, ICC of AIDR > 0.90, ICC of AiCE > 0.95, Talbe 4). Compared with FBP and DLR images, the calcified lesions were not identified in HIR images (Figure 2). In risk classification analysis, HIR reclassified one patient from moderate risk to low risk, five patients from high risk to moderate risk, and six patients from very high risk to high risk. In addition, DLR reclassified two patients from high risk to moderate risk and three patients from very high risk to high risk (Table 5). The weighted Kappa showed a value of 0.90 (95% CI, 0.85–0.96) between HIR and FBP, and 0.96 (95% CI, 0.92–0.99) between DLR and FBP, indicating better agreement between DLR and FBP in risk classification.

**Table 3:** The agatston score, calcium volume and mass of different reconstruction algorithms.

Measurement parameter	FBP	HIR	DLR	P
Calcium volume (mm^3^)	256.58 (60.66,349.19)	213.26 (51.12,290.76)	244.37 (58.72,319.55)	0.155
Agatston score	212.79 (74.00,398.06)	162.27 (49.10,291.25)	197.82 (62.93,344.85)	0.446
Calcium mass (mg/cm^3^)	34.14 (12.18,66.97)	22.40 (8.30,43.37)	31.61 (11.00,57.77)	0.069

Data are expressed as median (interquatile range).

**Table 4:** The intra- and inter-observer reproducibility of agatston score.

Method	FBP(ICC/*P* value)	HIR(ICC/*P* value)	DLR(ICC/*P* value)
First measurement vs. second measurement (Doctor A)	0.882/<0.001	0.940/<0.001	0.999/<0.001
First measurement vs. second measurement (Doctor B)	0.861/<0.001	0.946/<0.001	0.997/<0.001
Doctor A vs. doctor B (first measurement)	0.852/<0.001	0.924/<0.001	0.997/<0.001
Doctor A vs. doctor B (second measurement)	0.863/<0.001	0.908/<0.001	0.995/<0.001

**Table 5:** Effects of FBP, HIR, and DLR on agatston score-based risk classification.

Risk stratification based on FBP	Risk stratification based on HIR and DLR									
	HIR					DLR				
	Very low	Low	Moderate	High	Very high	Very low	Low	Moderate	High	Very high
Very low (*n* = 3)	3	0	0	0	0	3	0	0	0	0
Low (*n* = 14)	0	14	0	0	0	0	14	0	0	0
Moderate (*n* = 22)	0	1^a^	21	0	0	0	0	22	0	0
High (*n* = 39)	0	0	5^a^	34	0	0	0	2^a^	37	0
Very high (*n* = 26)	0	0	0	6^a^	20	0	0	0	3^a^	23
Total (*n* = 104)	3	15	26	40	20	3	14	24	40	23

FBP, filtered back projection; HIR, hybrid-iterative reconstruction; DLR, deep learning reconstruction.

^a^ Numbers indicated patients who changed risk classification with the use of HIR and DLR.

## Discussion

This study compared the performance of FBP, HIR, and DLR algorithms in image quality and CAC quantification. Our results indicated that DLR exhibited a remarkable enhancement of subjective image quality and objective metrics in comparison to FBP and HIR. In addition, DLR improved consistency of CAC quantification compared with FBP and HIR. DLR also reduced risk reclassification relative to HIR.

DLR algorithm utilizes deep neural network architectures trained on extensive datasets (19). Unlike conventional algorithms, DLR achieves self-learning capabilities to analyze image features, distinguish structural details from noise, and optimize image clarity through intelligent signal processing (19). Based on these methods, DLR has advantage for both the objective and subjective evaluation of coronary calcification. The results of this study were consistent with other studies which explored the advantage of DLR in optimizing imaging quality. Damiano Caruso et al. reported that comparable enhancements in the quality of coronary CTA images through the application of DLR led to improvements in both subjective and objective performance indicators (12). Cheng Xu et al. demonstrated that deep learning reconstruction algorithm achieved high image quality without changing the value of CT-derived fractional flow reserve (CT-FFR) (20). The results of our study expanded the application range to CAC and proved the DLR could enhance the image quality of CAC. In addition, our results demonstrated HIR algorithm significantly decreased the CT value and image noise of aortic root and left ventricle, however the DLR algorithm significantly reduced image noise without changing the CT value. Therefore, DLR algorithm does not influence the density of tissue which represents relevant disease or lesions in clinical diagnosis.

Ann-Christin Klemenz et al. reported that DLR results in less underestimation of CACS compared to ASiR-V when benchmarked against FBP, the conventional reference (21). Lijuan Zhu et al. reported that, compared to FBP, both DLR and ASiR-V improve CT image quality to different degrees (22). Yiran Wang et al. reported that no statistically significant different in calcium quantification between DLR and ASIR-V, despite the latter's improved image quality (23). All three articles performed risk stratification, calcium quantification, image quality, and utilized deep learning with deep convolutional neural networks. The results showed that the algorithms significantly improved image quality without affecting the consistency of diagnostic results, similar to the findings of this study. However, these three studies used DLR algorithm which was trained based on FBP images. In our research employed DLR algorithm which was trained based on MBIR images. Sandstedt Mårten et al. found that photo-counting CT quantified coronary calcifications more accurately than energy-integrating detector CT, but photon-counting CT is still in the early stages of clinical applications, and its widespread adoption faces the challenge of high equipment costs, while scanning protocols and post-processing standards need further improvement (24). Our study showed that DLR demonstrated the capability in the detection of microcalcifications, accurately pinpointing lesions which were not detectable by HIR (Figure 2). For calcium quantification, HIR yielded lower value of calcium volume, Agatston score and calcium mass than FBP and DLR, however the statistical analysis showed that the difference between HIR and FBP or HIR and DLR were no statistical significance (all *p* > 0.05). HIR missed calcification because HIR demonstrated significantly lower CT values in aortic root and left ventricle than DLR and FBP. In risk classification of coronary calcification, 12 cases showed changes in risk classification with the HIR algorithm compared to FBP, while only 5 cases exhibited such changes with DLR. Besides, 7 cases exhibited downgraded risk classification with HIR compared to DLR. Therefore, DLR reduced risk reclassification relative to HIR. Furthermore, patients whose risk classifications changed were predominantly concentrated in the high-risk and very high-risk groups. Therefore, as the burden of calcification escalated, the change of risk classification becomes evident. During automated calcium scoring with the software, because of the high image noise, FBP algorithm exhibited a tendency to misidentify non-calcified regions as calcified plaques (Figure 2).

This study has some limitations. Firstly, the single-center design and moderate sample size (*n* = 104) limit the generalizability of our results, especially for the pre-specified subgroup analysis, such as the high-risk population vs. the medium- to low-risk population. Therefore, future studies should validate our findings in larger multi-center cohorts to ensure broad applicability in clinical settings. Secondly, this study validated the DLR algorithm using only one coronary artery calcium scoring software, future research could extend to analysis with multiple calcification scoring platforms.

In conclusion, compared with traditional FBP and HIR, DLR enhances the image quality and consistency of CAC quantification. Besides, DLR reduced risk reclassification relative to HIR.

## Data Availability

The raw data supporting the conclusions of this article will be made available by the authors, without undue reservation.
